# Comparison of DEM Super-Resolution Methods Based on Interpolation and Neural Networks

**DOI:** 10.3390/s22030745

**Published:** 2022-01-19

**Authors:** Yifan Zhang, Wenhao Yu

**Affiliations:** 1 School of Geography and Information Engineering, China University of Geosciences, Wuhan 430074, China; YifanZhang@cug.edu.cn; 2 National Engineering Research Center for Geographic Information System, China University of Geosciences, Wuhan 430074, China

**Keywords:** DEM, super-resolution process, neural network, terrain features

## Abstract

High-resolution digital elevation models (DEMs) play a critical role in geospatial databases, which can be applied to many terrain-related studies such as facility siting, hydrological analysis, and urban design. However, due to the limitation of precision of equipment, there are big gaps to collect high-resolution DEM data. A practical idea is to recover high-resolution DEMs from easily obtained low-resolution DEMs, and this process is termed DEM super-resolution (SR). However, traditional DEM SR methods (e.g., bicubic interpolation) tend to over-smooth high-frequency regions on account of the operation of averaging local variations. With the recent development of machine learning, image SR methods have made great progress. Nevertheless, due to the complexity of terrain characters (e.g., peak and valley) and the huge difference between elevation field and image RGB (Red, Green, and Blue) value field, there are few works that apply image SR methods to the task of DEM SR. Therefore, this paper investigates the question of whether the state-of-the-art image SR methods are appropriate for DEM SR. More specifically, the traditional interpolation method and three excellent SR methods based on neural networks are chosen for comparison. Experimental results suggest that SRGAN (Super-Resolution with Generative Adversarial Network) presents the best performance on accuracy evaluation over a series of DEM SR experiments.

## 1. Introduction

As one of the most important digital representations of terrain, DEMs record spatial elevation information in a regular raster form [1]. Through visualizing fluctuating characters of terrain surfaces, DEMs can be widely applied in the domains including facility siting, hydrological analysis, and urban design [2,3,4]. With the rapid development of measuring equipment, DEM data can be generated from various sources, which accelerate its universality in landform analysis applications [5]. Specifically, the synthetic aperture radar (SAR) has been used as the primary source of DEMs at a global scale for its robustness in all weather conditions [6]. Despite the wide usage of SAR data, the limitation of equipment precision can still result in systematic errors that reduce the resolutions of DEM products. The inadequate spatial resolution of DEM data also restricts its usage in terrain-related analyses [7,8]. For example, using recent and accurate topographic data rather than low-resolution DEM data can obtain better accuracy for flood inundation modeling [9]. In addition, higher-resolution DEM is able to return more accurate predicted terrain features such as stream network and sub-basin classification [10]. The most direct solution to obtain high-resolution DEM is to improve the precision of measuring equipment, but this process is difficult, costly, and time-consuming. Therefore, generating high-resolution DEMs without extra cost becomes a key concern of researchers from various fields [11,12].

Traditionally, a practical way to obtain high-resolution DEMs without extra cost is to recover from easily obtained low-resolution DEMs, and this process is termed DEM super-resolution (SR) [12]. There are two major solutions for DEM SR, and the first solution is to generate a high-resolution DEM from multiple low-resolution DEMs of the same scene [13]. For example, Yue et al. [14] proposed a regularized SR method for DEM SR. In this method, a wider and higher-resolution DEM can be obtained by the fusion of DEMs with different spatial coverage, resolution, and accuracy. Different from the first solution, the second is the single DEM SR, in which a high-resolution DEM can be generated by only using one source of the corresponding low-resolution DEM. However, priori knowledge or pre-training is usually needed [11]. For example, based on the priori knowledge that nearer objects are more similar, spatial interpolation (e.g., bicubic interpolation) is commonly used in the single DEM SR.

Although the first type of method can improve DEM resolution, it requires extra topographic information, which is still hard to acquire in practical applications, especially for a large area [15]. Therefore, the second solution is more adaptable for most application scenarios, which is also the concern of this paper. Nowadays, with the rapid development of machine learning, neural network methods give more possibility to solve difficulties that used to have rare solutions [16], and there has been observed great progress in the field of computer vision, especially for image SR [17,18]. For both the image SR and DEM SR, the target is to improve the quality of field data from its blurred and down-scaled noisy version [19]. Compared with traditional image SR methods, neural network SR methods can greatly improve the quality of the result using a learning strategy [20]. Moreover, the “end-to-end” architecture makes it easier for neural networks to learn the complex restoration process in SR [21].

It is inspiring that the neural network SR methods are effective for various types of images, e.g., human faces, flowers, and animals [22,23]. However, whether these methods are suitable for DEM SR is uncertain, considering that DEM data present much more complex terrain characters (e.g., peak, saddle, and valley) than natural images [24,25]. In addition, pattern recognition for images (e.g., faces) is based on features, not on continuous surfaces such as in DEMs. Moreover, the significant differences between the terrain elevation field and the image RGB field increase the difficulty of generating high-resolution DEMs from a low-resolution one. The elevation field also usually has a much larger range than the image RGB field. In addition, current image SR methods focus more on the perceptual feeling of generated images [26], and this is different from the target of DEM SR, in which the accuracy and terrain features of generated DEM are more concerned [27].

Some related pioneering studies suggested the huge potential to apply neural network methods to DEM data [28]. However, the evaluation and comparison of these methods on the task of DEM SR are urgently needed. Therefore, this paper investigates the effectiveness of image SR methods on terrain data from a comprehensive perspective. More specifically, the interpolation method (bicubic interpolation) and three excellent SR methods based on neural networks are chosen for comparison. Among them, CEDGAN [28] is designed for spatial interpolation, which has already been proved to be effective for DEM data. ESRGAN [18] is an improved version of SRGAN [17], which focuses more on the perceptual feeling of generated images, and its results are suggested to be more realistic [18]. Generally, both SRGAN and ESRGAN are accepted to be outstanding image SR methods [29]. In this paper, indexes related to terrain features including elevations and terrain derivatives were compared to evaluate the effectiveness and suitability of these methods.

The remainder of this paper is organized as follows. Section 2 reviews the related work of SR methods. Section 3 describes the interpolation method and three outstanding DEM SR methods in detail. Section 4 introduces the design of the experimental evaluation and presents results and discussions. Section 5 presents conclusions and discusses future work.

## 2. Related Work

SR is one of the key concerns in the field of computer science, which aims to generate a high-resolution image based on a single or several low-resolution images. Since it was first proposed [30], researchers from various fields proposed many relevant methods, which are usually classified into three categories: interpolation-based, reconstruction-based, and learning-based [11]. Firstly, interpolation-based methods improve the resolution of low-resolution images by estimating values of unknown pixels in target high-resolution images [31]. In this process, the interpolation kernel (or function) is the key concern; given a specific interpolation kernel, values of unknown pixels can be calculated from their neighboring pixels. However, although interpolation-based methods are less time consuming, they tend to blur high-frequency regions during the estimation. Then, to tackle this problem, reconstruction-based methods integrate image prior knowledge to generate high-resolution images such as gradient profile prior [32] and natural image prior [33]. Therefore, compared with the interpolation-based methods, the reconstruction-based methods have the advantages of preserving edges and suppressing artifacts. However, this type of method is not suitable to produce high-resolution images at relatively large magnification factors. Finally, considering the learning-based strategies, the relevant methods attempt to construct the direct relations between high-resolution images and low-resolution images. For example, inspired by the idea of compress sensing, Yang et al. [19] proposed to jointly learn two dictionaries for both high-resolution images and low-resolution images. In this way, high-resolution images can be reconstructed by the sparse codes of low-resolution images and the corresponding high-resolution dictionary. Although learning-based methods can gather high-frequency information from a learning process, the quality of training data will have a great impact on results [34].

DEM SR techniques developed along with the road of image SR, and the three types of image SR methods can be also applied to DEM SR [35,36]. However, similar dilemmas of these methods in image SR have also appeared in DEM SR. Then, researchers attempt to solve this problem from other perspectives, and the development of neural networks brings a new direction. For example, Dong et al. [21] introduced convolutional neural network (CNN) for image SR, which learns an end-to-end mapping between low-resolution images and high-resolution images. In this method, a lightweight but effective network structure is adopted, which shows better overall image quality. Then, presenting the potential to solve undetermined problems, generative adversarial networks (GANs) have also been applied to image SR. For example, Ledig et al. [17] proposed a single image SR method based on a generative adversarial network (SRGAN), which obtained effective results on the perceptual feelings of images. Then, by improving the network structure of SRGAN, Wang et al. [18] proposed ESRGAN to further enhance the visual quality of generated images. Based on the performance of image SR methods, some researchers have also applied these methods to DEM data [34]. However, the recent image SR methods based on neural networks mainly focus on the visual quality of high-resolution images, while the focus of DEM SR is more concerned with the accuracy and terrain features of generated high-resolution DEM. Therefore, this paper aims to investigate whether image SR methods with the target of visual quality are suitable for the task of DEM SR. More specifically, three excellent SR methods based on neural networks (including SRGAN, ESRGAN, and CEDGAN) and the commonly used interpolation method (bicubic interpolation) are chosen for comparison. Details of these techniques are presented in Section 3.

## 3. Methods

### 3.1. SR Methods Based on Interpolation

**Figure 1:**
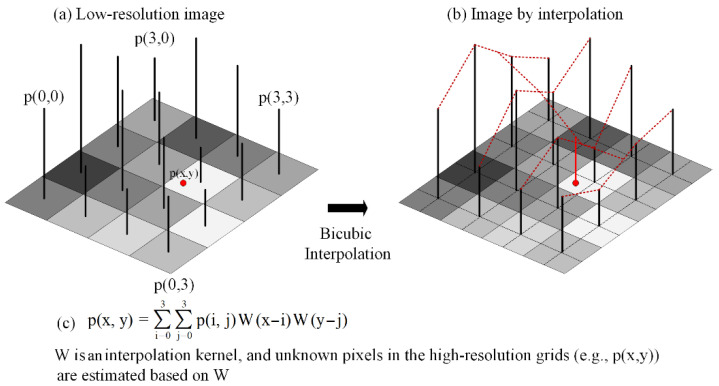
The process of bicubic interpolation.

Interpolation SR methods generate high-resolution images by estimating unknown pixel values in the high-resolution grids [31,37]. In this process, the value of an unknown pixel is calculated by its neighboring pixels based on a specific interpolation kernel. The commonly used interpolation methods include nearest neighbor interpolation, bilinear interpolation, and bicubic interpolation, among which the bicubic interpolation obtains the best accuracy [38]. Therefore, in this paper, bicubic interpolation is chosen for comparison, and this interpolation process is presented in Figure 1. In the bicubic interpolation, values of unknown pixels in the high-resolution grids (e.g., *p*(*x*,*y*)) are estimated based on their nearest 16 pixels, and the formula is presented in Figure 1c. From this formula, high-frequency details will be easily smoothed for estimating unknown pixel values by the weighted sum of neighboring pixel values. Moreover, the accuracy of the interpolation results depends on the interpolation kernel W. Therefore, for valid comparisons, a commonly used interpolation kernel is chosen in this paper, which is described as Equation (1).

(1) $$W\left(t\right)=\{\begin{array}{c} \left(a+2\right){\left|t\right|}^{3}-\left(a+3\right){\left|t\right|}^{2}+1\;\left|t\right|<1\\ a{\left|t\right|}^{3}-5a{\left|t\right|}^{2}+8a\left|t\right|-4a\;1<\left|t\right|<2\\ 0\;otherwise \end{array}$$

where t denotes distances between unknown pixels (e.g., *p*(*x*,*y*)) and existing pixels, and *a* is a given parameter, which is usually set as −0.5.

### 3.2. SR Methods Based on Neural Networks

**Figure 2:**
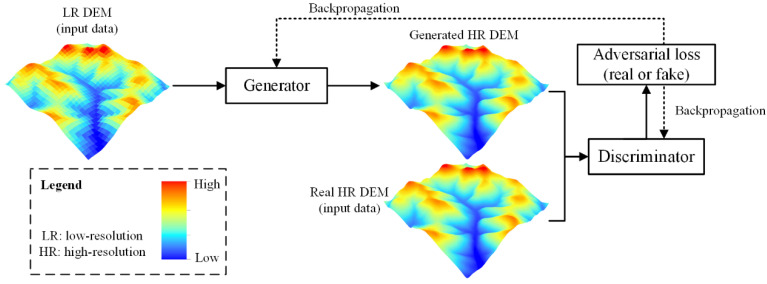
A general structure of SR model based on GAN.

Recently, neural networks have been introduced to various tasks of computer vision including image segmentation, image classification, and image SR [39,40,41]. Compared with traditional image SR methods (e.g., sparse reconstruction), neural network SR methods can learn an end-to-end mapping between high-resolution (HR) and low-resolution (LR) images, and complex features can be automatically learned by hidden layers. Therefore, the pattern of hidden layers is critical to final results, for which most concerns have been paid to the structure design of neural networks. Among the neural network structures, generative adversarial networks (GANs) adopt a unique adversarial structure, which is suggested to have the advantage of sidestepping the difficulty of approximating many intractable probabilistic computations [42]. More specifically, there are two independent neural networks in GAN, which include a generator (*G*) and a discriminator (*D*). During the training process, the discriminator is trained to make the best judgment, while the generator is trained to maximally confuse the discriminator. When applied to image SR, the discriminator is trained to distinguish whether an image is real or generated by the generator, while the generator is trained to generate fake realistic images that the discriminator cannot distinguish. Therefore, with such a training strategy, when a training balance is reached, the generator can automatically learn to generate high-resolution images that are highly similar to the real images. This training process can be described as Equation (2).

(2) $$\underset{\theta_{G}}{\min}\underset{\theta_{D}}{\max}E_{I^{HR}\sim p_{train}\left(I^{HR}\right)}\left[\log D_{\theta_{D}}\left(I^{HR}\right)\right]+E_{I^{LR}\sim p_{G}\left(I^{LR}\right)}\left[\log(1-D_{\theta_{D}}\left(G_{\theta_{G}}(I^{LR}\right))\right]\;$$

where $\log D_{\theta_{D}}\left(I^{HR}\right)$ denotes that the discriminator (*D*) can correctly recognize real high-resolution images ($I^{HR}$), and $\log(1-D_{\theta_{D}}\left(G_{\theta_{G}}(I^{LR}\right))$ denotes that the discriminator (*D*) can correctly recognize fake images generated by the generator ($G_{\theta_{G}}(I^{LR})$). A general framework of this training process is presented in Figure 2. The adversarial loss is obtained from the discriminator, which can be automatically calculated during the process of adversarial training. This type of loss function is also a unique characteristic of GAN, which means that GAN can be trained without other specifically designed loss functions. Therefore, the design of the generator and discriminator has a large impact on the final results, which is also the major concern of scientists from many fields.

Nowadays, there are some pioneering works that introduce GAN to the task of SR, and the variations of the model include SRGAN and ESRGAN. A similar structural feature of these models is to input LR (low-resolution) images and output HR (high-resolution) images that preserve similar global data distribution patterns with the corresponding LR images. This feature is also suitable for DEM data. Then, CEDGAN is specifically designed for spatial interpolation, and experiments in terms of DEM data present that CEDGAN is more effective than traditional interpolation methods (e.g., kriging interpolation) [28]. Therefore, in this paper, we will investigate their applicability to DEM SR in a systematic manner. Details of their structures and advantages are discussed in the following sections.

#### 3.2.1. SRGAN

**Figure 3:**
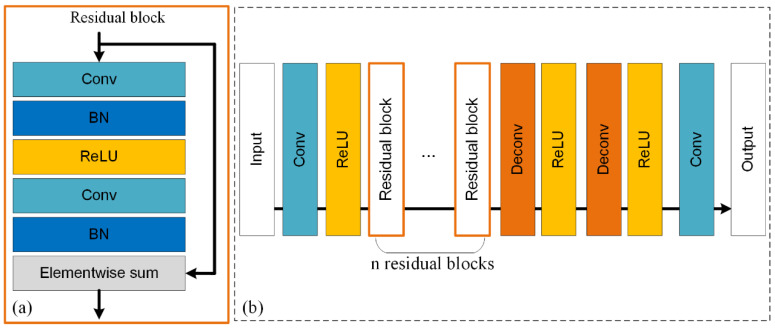
(**a**) Residual block and (**b**) the architecture of generator in SRGAN (Conv: convolution, BN: batch normalization, ReLU: rectified linear unit, Deconv: deconvolution).

The rise of convolutional neural networks (CNNs) had a substantial impact on image SR, which greatly enhanced the accuracy of image SR. Before SRGAN, most image SR methods based on neural networks are of supervised learning, the optimization target of which is usually the minimization of the mean squared error (MSE) between recovered images and corresponding real HR images. Although it is convenient for optimizing, the training index of MSE is unable to capture perceptual features of images (e.g., high-frequency texture detail), as it is defined based on pixel-wise image differences. Therefore, Ledig et al. [17] firstly attempted to integrate GAN to improve the photo-realistic perception during image SR, and a method named SRGAN is proposed. In this approach, a perceptual loss function is well designed to replace the original commonly used pixel-based loss function. There are two major parts in the perceptual loss function, i.e., adversarial loss and content loss. Different from the most widely used pixel-based content loss (MSE), a VGG loss, which is based on the ReLU activation layers of the pre-trained 19 layer VGG network presented in [43], is defined to describe the content loss. In other words, high-level features of real HR images and generated images from the generator are firstly extracted, and then, the Euclidean distance between the two feature representations is calculated as the content loss. In this way, more and finer perceptual texture features can be perceived during the training process, which can be ultimately recovered on the generated images. Details of the generator in SRGAN are presented in Figure 3. It can be observed that the residual block with an identical layout is the major core of the generator. The residual block is proposed by [44], which is proposed to solve training problems of deep neural networks. More specifically, deep neural networks are difficult to train, and the performance will degrade when stacking excessive layers. With the residual block, features extracted by shallower layers can be effectively transmitted to corresponding deeper layers. This advantage is significant for image SR, since feature extraction is critical to the final generated results.

Therefore, compared with other SR methods based on neural networks, SRGAN can train the generator to perceive more perceptual features and generate more photo-realistic images. Compared with images, there are some common features in DEM data. For example, characters of terrain textures on plain areas are similar to image textures, as they both present smooth transitions. However, as mentioned above, there are also some unique terrain texture features in DEM data (e.g., ridge and river), which is very different from the natural texture features in images. Moreover, the elevation differences in neighboring cells are significant in many complex terrain areas (e.g., bluff areas), and that is much larger than the largest difference between pixel values (i.e., the range of RGB value of 0–255). Therefore, whether this natural image texture features-based method is suitable for DEM SR task needs to be carefully explored, and more details of experiments are presented in Section 4.

#### 3.2.2. ESRGAN

**Figure 4:**
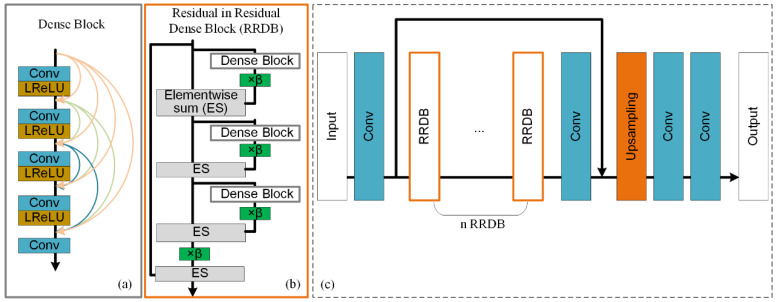
(**a**) Dense block, (**b**) Residual in Residual Dense Block (RRDB), and (**c**) the architecture of generator in ESRGAN (LReLU: Leaky ReLU, β: residual scaling parameter).

ESRGAN is proposed based on SRGAN, which is suggested to improve the overall perceptual quality of SR images [18]. In ESRGAN, there are two major modifications in the generator, which are presented in Figure 4. The first modification is to remove all Batch Normalization (BN) layers (Figure 4a), and the second is to replace the original residual block with the proposed Residual-in-Residual Dense Block (RRDB, Figure 4b). Firstly, in SRGAN, features extracted from former layers (e.g., convolution) will be normalized by the BN layer using the mean and variance of each batch during training, and using the estimated mean and variance of the whole training dataset during testing. In this way, Wang et al. [18] suggest that BN layers will bring unpleasant artifacts and limit the generation ability when there are huge differences between the statistics of training and testing datasets. Therefore, BN layers are removed in this method. Then, based on the observation that more layers and connections could always boost performance, the original residual block in SRGAN is replaced by RRDB, which employs a deeper and more complex structure (Figure 4b). With such two modifications, ESRGAN is suggested to be capable of generating more realistic textures during image SR than SRGAN. Therefore, this paper aims to investigate whether ESRGAN has a similar better performance than SRGAN on DEM SR, and more details of experiments are presented in Section 4.

#### 3.2.3. CEDGAN

**Figure 5:**
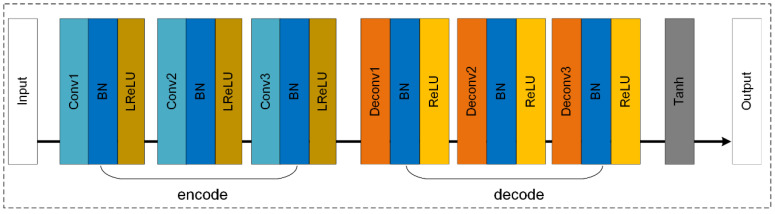
The architecture of generator in CEDGAN.

CEDGAN is specially designed for spatial interpolation, and that is an endeavor to investigate the deep spatial knowledge using artificial intelligence [28]. Different from the above two methods, this method introduces the structure of conditional generative adversarial networks (cGAN), which is an extension of GAN [45]. In this method, an encoder–decoder structure is used to construct the generator, which is suggested to be suitable for spatial feature extraction [46], and the structure of the generator is presented in Figure 5. It can be observed that compared with SRGAN or ESRGAN, the generator in CEDGAN is a lightweight network, and fewer parameters make it easier to train. The experimental results in Zhu et al. [28] present that with only several iterations, relatively accurate DEMs can be generated by the generator. Then, with more iterations, this method can generate more accurate DEMs than traditional spatial interpolation methods (e.g., the kriging method). Therefore, this specially designed method is also chosen to compare with image SR methods to show which is better for DEM SR, and more details of experiments are presented in Section 4.

### 3.3. Evaluation Indexes

Considering terrain features and the realistic demand of high-resolution DEM data, several commonly used evaluation indexes related to the accuracy and features of DEM are chosen for comparison, and they can be represented in general root mean square error (*RMSE*) and mean error (*ME*) forms presented in Equation (3).

(3) $$\begin{array}{c} RMSE_{F}=\sqrt{\frac{\sum_{i=1}^{n}{\left(F_{oi}-F_{gi}\right)}^{2}}{n}}\\ ME_{F}=\frac{\sum_{i=1}^{n}\left|F_{oi}-F_{gi}\right|}{n} \end{array}$$

where $F_{oi}$ and $F_{gi}$ denote the attribute value of feature *F* (slope, aspect, and elevation) of the *i*th cell in the original real HR DEM and generated fake HR DEM, respectively, *n* is the total cell number of DEM, and $ME_{F}$ denotes the mean error of feature *F*. In this paper, three types of features including elevation, slope, and aspect are chosen. Among these indexes, the *RMSE* of elevation can reveal the global accuracy of generated DEMs, and features of slope and aspect can reveal the extent of feature preservation of generated DEMs. In addition to the evaluation of terrain structural features, the evaluation of terrain critical points is also important [47]. Therefore, the displacement distances of peak points and valley points between real HR DEM and generated HR DEM are used to evaluate the ability of the methods to preserve terrain critical points. In our experiments, the top 20 peak points and the lowest 20 valley points are chosen to calculate their displacement distances, and a shorter distance represents a better feature preservation performance.

## 4. Experiments and Results

### 4.1. Data Descriptions and Parameters

**Figure 6:**
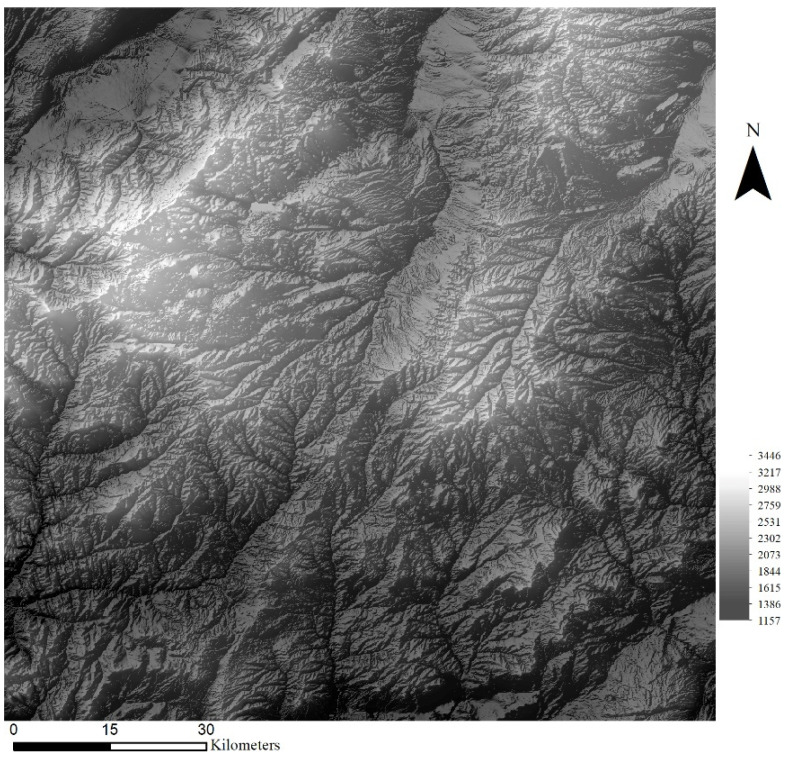
An example region of the DEM data (3584 × 3584).

To investigate the effectiveness of the selected methods on DEM SR (SRGAN, ESRGAN, and CEDGAN), a dataset of digital elevation models (DEMs) with complex terrain features is used in this paper, which was acquired from the USGS (United States Geological Survey). More specifically, DEM data with the size of 3584 × 3584 from ten different regions are selected, and terrain elevations in the dataset range from 0.5 to 3741 m. The resolution of DEM data is 30 m, and an example region is presented in Figure 6. There can be observed many typical terrain features such as ridges, rivers, and mountains, and there are also some discrete areas with sudden elevation changes, which are much more complex than texture details in natural images. After preprocessing, single-channel DEM tiles (1 × 64 × 64) with no repetition are randomly cropped, and a total of 31,360 high-resolution DEM tiles are obtained. To address the concerns of over-fitting and memorization of training samples, a method of systematic sampling is used to sample 25,088 DEM tiles as training data and the other 6272 DEM tiles as validation data. More specifically, an equal number of DEMs will be sampled from every region. To accommodate the selected methods to DEM data, the original three-channel network structures in SRGAN and ESRGAN are adapted for single-channel data (CEDGAN is a single-channel network). Moreover, DEM tiles are normalized into float tensor units ([−1.0,1.0]) based on the respective maximum and minimum cell values of each DEM [28], and this process is presented in Equation (4):

(4) $${\mathrm{DEM}}_{i-n}=2\times \frac{{\mathrm{DEM}}_{i}-H_{min}}{H_{max}-H_{min}}-1\;$$

where ${\mathrm{DEM}}_{i-n}$ denotes the normalized version of ${\mathrm{DEM}}_{i}$, and $H_{max}$ and $H_{min}$ denote the maximum and minimum cell values of ${\mathrm{DEM}}_{i}$, respectively. In this way, the large elevation gaps between DEM tiles from different areas (e.g., depression and plateau) can be avoided, and tiny terrain features can be learned rather than overlooked during the training process. That can help with terrain features recovery after DEM SR. Finally, all the elevation values are mapped back to their original values in the reported accuracies, and the corresponding evaluation indexes can be calculated based on the obtained back-mapped DEMs. The data that support the findings of this study are available in [figshare.com] with the identifiers (https://figshare.com/s/3fe0b1313938b0db994a (accessed on 13 May 2021)).

Then, some parameters also need to be specified before the training process. Most of the parameters (e.g., optimizer, the slope of the leak for layers with LeakyReLU activation) are kept the same as their original setting in the above methods for performance comparisons. Then, the learning rates of different methods are tuned for the best training results (0.0000001 in SRGAN, 0.0002 in ESRGAN, and 0.00001 in CEDGAN). All experiments are conducted with a downsampling factor of 4, and LR DEMs are obtained by downsampling the HR DEMs using a bicubic kernel with the same downsampling factor. All the models are trained with 100 epochs, and the training images are divided in a random way in each epoch.

### 4.2. Training Procedure

**Figure 7:**
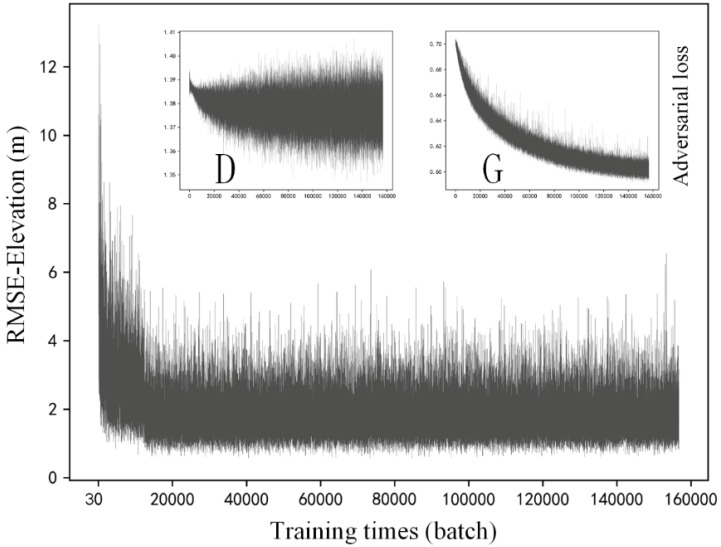
Training details of SRGAN: variation of the model accuracy (RMSE-Elevation) and adversarial losses of generator (G) and discriminator (D) during the training procedure.

**Figure 8:**
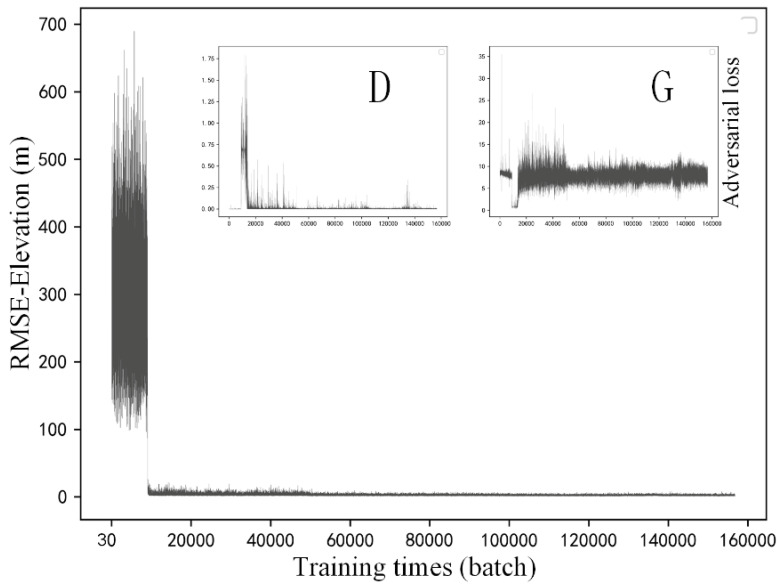
Training details of ESRGAN: variation of the model accuracy (RMSE-Elevation) and adversarial losses of generator (G) and discriminator (D) during the training procedure.

**Figure 9:**
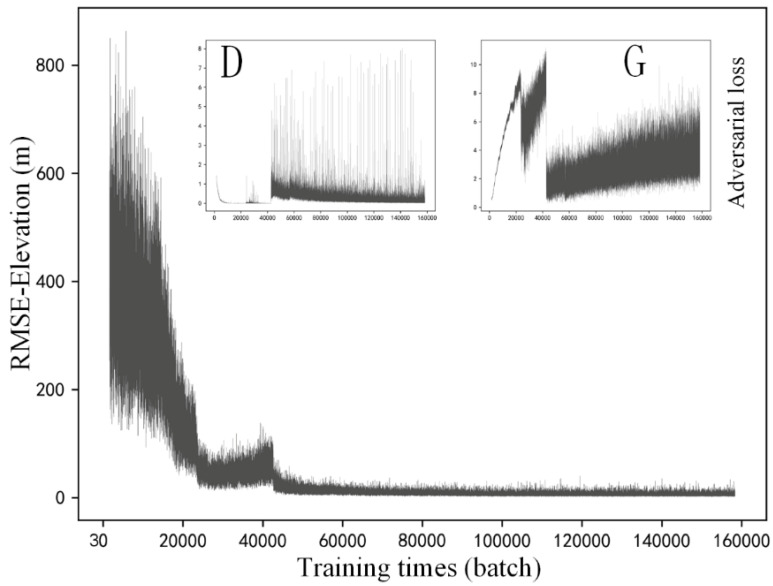
Training details of CEDGAN: variation of the model accuracy (RMSE-Elevation) and adversarial losses of generator (G) and discriminator (D) during the training procedure.

In this section, training details of the selected methods are described. Figure 7, Figure 8 and Figure 9 respectively present the variation of model accuracy (RMSE-Elevation) and adversarial losses for generator (G) and discriminator (D) in SRGAN, ESRGAN, and CEDGAN during the training procedure. Firstly, as for SRGAN (Figure 7), values of the model error drop dramatically at the first 20,000 batches and become stable after that. We train on 156,800 batches (100 epochs), and it presents that the average error of generated high-resolution DEM gradually stabilized at 1.7 m, which is a surprising result considering complex DEM training data and challenging parameters setting including the relatively larger patch size (1 × 64 × 64) and downsampling factor (4×) used in our training process. Then, the average errors of ESRGAN and CEDGAN are 2.2 meters and 6.4 m, respectively. However, the bicubic interpolation method can obtain an average error of 2.1 m on training data without any pre-training. Therefore, it can be observed that only SRGAN outperforms the bicubic interpolation method during the training process.

In addition to values of accuracy during the training process, adversarial losses for D and G of these methods are also described at the upper location in Figure 7, Figure 8 and Figure 9. Firstly, in Figure 7, there can be observed a decreasing tendency of both adversarial losses, which reveals an intrinsic characteristic of GAN (that is, the adversary system results in the development of both D and G). A similar phenomenon appears in the adversarial losses in ESRGAN and CEDGAN (Figure 8 and Figure 9), but their competitions present to be more intense than that in SRGAN. For example, in CEDGAN, the loss of D decreased, and the loss of G increased in the first 20,000 training batches, and that means D suppresses G. Then, the competition state reverses after some training batches (i.e., G is stronger than D). Finally, both D and G gradually become stable. Therefore, it can be concluded that the three methods tend to converge to their game equilibrium during the training.

### 4.3. Results

Although these methods present different performances on training data, their ability to generate high-resolution DEMs based on the validation set is more focused. Therefore, in this section, we apply the three well-trained models on the validation set to test whether they are over-fitting or memorize training samples. Three types of evaluation are proposed on these methods.

#### 4.3.1. Quantitative Evaluation

In this section, the evaluation indexes proposed in Section 3.3 are utilized to test the effectiveness of the three well-trained models. The validation set consists of 6272 DEM tiles from ten different regions, and the evaluated results of the generated high-resolution DEMs are presented in Table 1. It should be noted that these evaluation index values of all DEM tiles are respectively averaged for a global evaluation. From Table 1, the main observation is that the three neural network-based methods (SRGAN, ESRGAN, and CEDGAN) generate worse results in terms of RMSE-Elevation on the validation set than those on the training data, and that is also a characteristic of learning methods. Nevertheless, the results are still acceptable with small floating precision, and thus, our training is effective. In addition, SRGAN outperforms the other three methods in terms of the indexes of RMSE-Elevation, RMSE-Slope, and RMSE-Aspect. However, considering the preservation of critical points (i.e., peak and valley points), bicubic interpolation (Bicubic) obtains the best results. This is caused by the difference between DEM generation mechanisms of interpolation-based methods and neural network-based methods. More specifically, for interpolation-based methods, some cell values are kept fixed, and only unknown cell values are estimated in terms of existing cell values; as a comparison, all cell values will be regenerated by neural network-based methods. In other words, there is a global restriction framework in interpolation-based methods, which makes constraints for peak and valley points (please see Figure 1). Then, as for neural network-based methods, SRGAN still obtains the best results.

**Figure 10:**
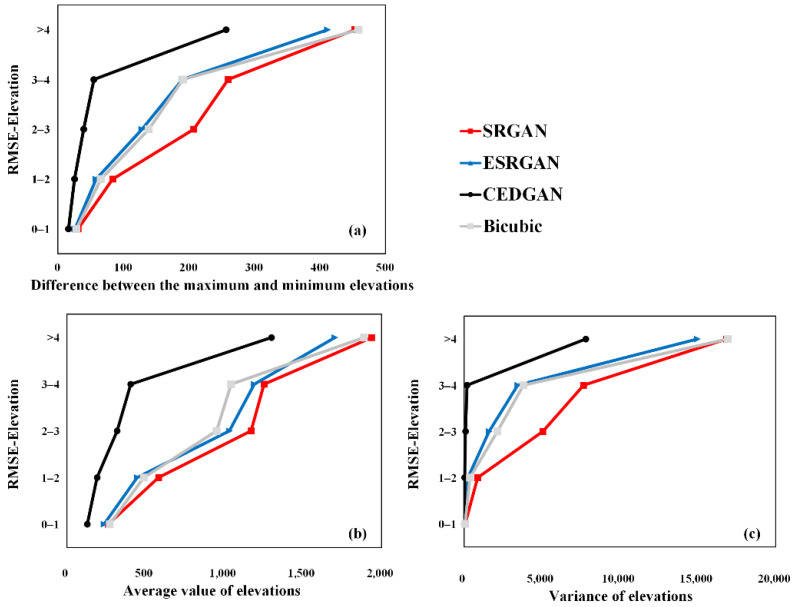
The correlation between terrain complexity and DEM SR precision (RMSE-Elevation): (**a**) difference between the maximum and minimum elevations; (**b**) average value of elevations; (**c**) variance of elevations.

Next, more details of the performance of these methods on the validation set are investigated. Firstly, the range of the obtained results of RMSE-Elevation by these methods is divided into five grades (i.e., 0–1, 1–2, 2–3, 3–4, and larger than 4). Then, features of terrain elevations on the corresponding real high-resolution DEM tiles in each grade are analyzed. More specifically, if a method generates a fake high-resolution DEM with the RMSE-Elevation larger than 4, the corresponding real high-resolution DEM will be grouped into “>4” (please see Figure 10). Then, three indexes including the elevation difference between the maximum and minimum, average value of elevations, and variance of elevations of the high-resolution DEM tile are calculated to reveal its terrain complexity; and the values of the three indexes of all DEM tiles in the group “>4” are respectively averaged to reveal global characteristic. The results are presented in Figure 10. It can be first observed a strong correlation between the RMSE-Elevation and terrain complexity. That means DEMs with more complex terrain features (i.e., higher values of the three indexes) are more difficult to recover (larger errors) during the SR process. Then, SRGAN presents to be the most robust method to generate high-resolution DEM, for which SRGAN can recover LR DEM tiles with the same level of terrain complexity to relatively higher precision. Therefore, it can be concluded that SRGAN is better than the other three methods for DEM SR considering both the robustness and preservation of accuracy and features on the generated high-resolution DEMs.

#### 4.3.2. Visual Evaluation

**Figure 11:**
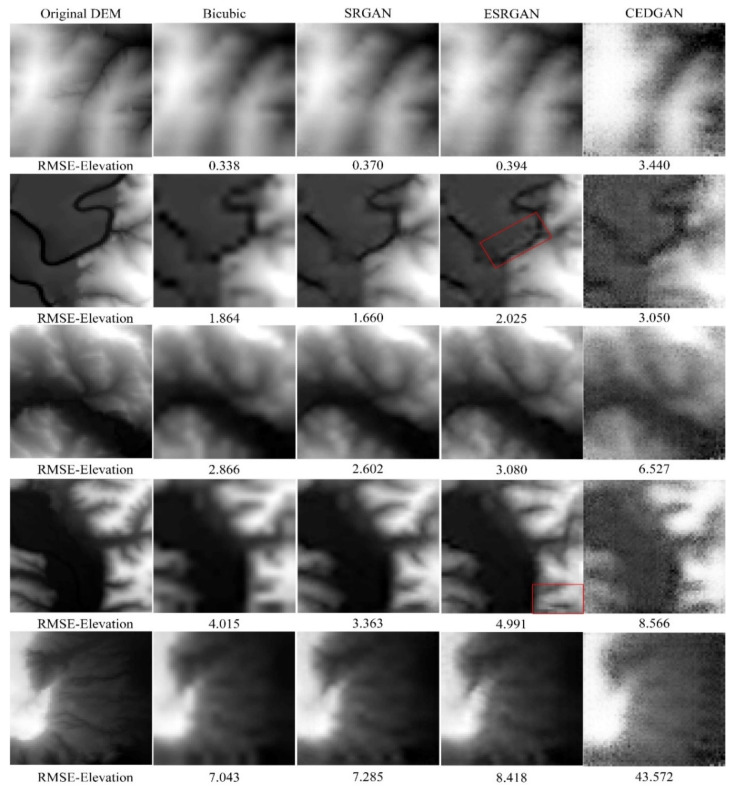
The super-resolution results of the selected methods (×4).

Although SRGAN outperforms the other methods from the perspective of quantitative evaluation, visual evaluation is needed, as there are many cases in image SR in which images with better quantitative indexes present worse visual perception [17,18]. In this section, a visual evaluation of these methods is implemented, and we selected from each grade a representative DEM tile for illustrating the results (Figure 11). From Figure 11, it can be observed that terrain textures are always over-smoothed in the results of bicubic interpolation. Therefore, although bicubic interpolation outperforms ESRGAN in terms of the index of RMSE-Elevation, the results of ESRGAN present a comparable (or better) visual perception than bicubic interpolation (i.e., preserving more terrain details). However, some local terrain textures seem to be distorted in the recovered DEMs by ESRGAN (red boxes in Figure 11), which might be the factor to reduce its accuracy. As a comparison, this phenomenon rarely appears in the results of SRGAN, and thus, SRGAN outperforms the other methods from this perspective. Finally, considering CEDGAN, there are some noisy signals in its results, and those seem to be much worse than the other methods. Therefore, the visual results suggest that CEDGAN may not be appropriate for the task of DEM SR.

#### 4.3.3. Evaluation of Terrain Features Preservation

Finally, we investigate the effectiveness of the methods in recovering terrain features. From the previous evaluations, it can be observed that CEDGAN generates the worst results. Therefore, in this evaluation, only bicubic interpolation, SRGAN, and ESRGAN are focused, and two DEM tiles in two accuracy grades with typical terrain features are chosen for comparison.

**Figure 12:**
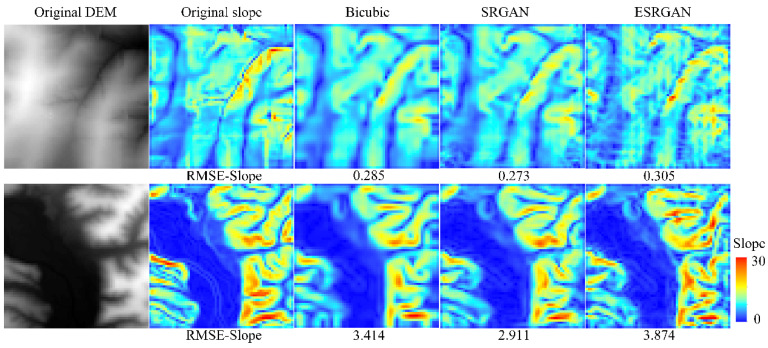
Slope results of the selected methods.

**Figure 13:**
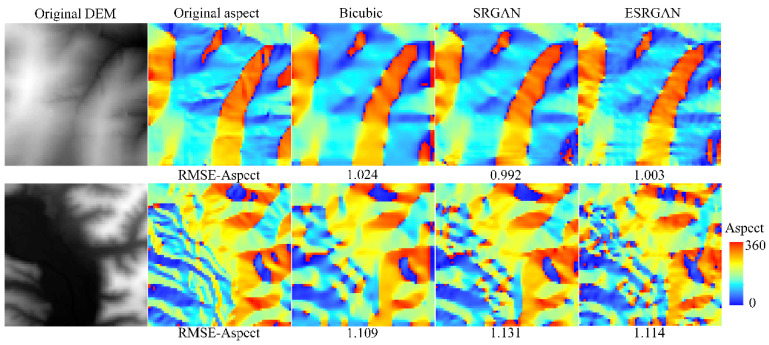
Aspect results of the selected methods.

Figure 12 and Figure 13 present the slope and aspect results of the original DEMs and the high-resolution DEMs generated by these methods, respectively. Considering the evaluation of slope, intuitive perception is that the results of bicubic interpolation are much smoother than those of SRGAN and ESRGAN, and some textures disappear in the results of bicubic interpolation but are retained in SRGAN and ESRGAN. Then, compared with SRGAN, the slope results of ESRGAN present a much more complex feature distribution pattern (such as grid-line distribution). In the computer vision field, the advantage of ESRGAN is that it can preserve more local perceptual features with a more complex network structure when dealing with natural images [18] (see Figure 4). However, it can be observed from the slope results that for DEM SR, without the constraint of global structure (the global precision RMSE-Elevation), local features generated by ESRGAN also present to be out of control. Intuitively, distortions in the results of ESRGAN are obvious compared with the results of SRGAN and bicubic interpolation. However, the slope results of SRGAN present to keep a better balance between global precision and local features, which may be the reason why SRGAN outperforms the other methods. Then, considering the evaluation of aspect, the three methods generate comparable results, and such a conclusion is consistent with the quantitative evaluation in Table 1.

**Figure 14:**
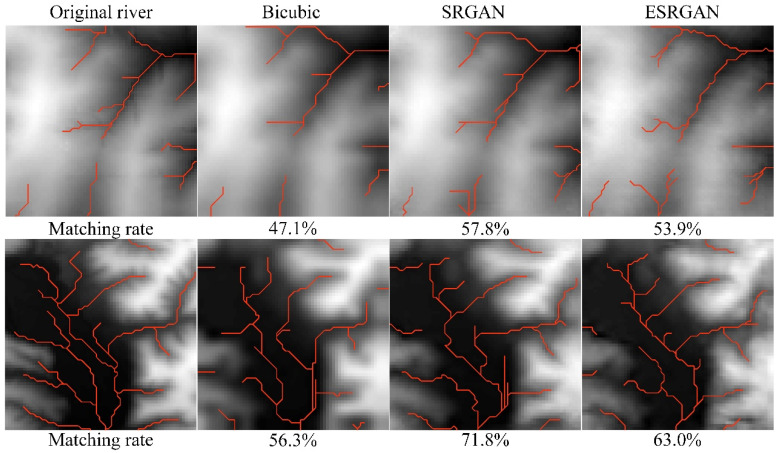
Stream matching results of the selected methods.

**Figure 15:**
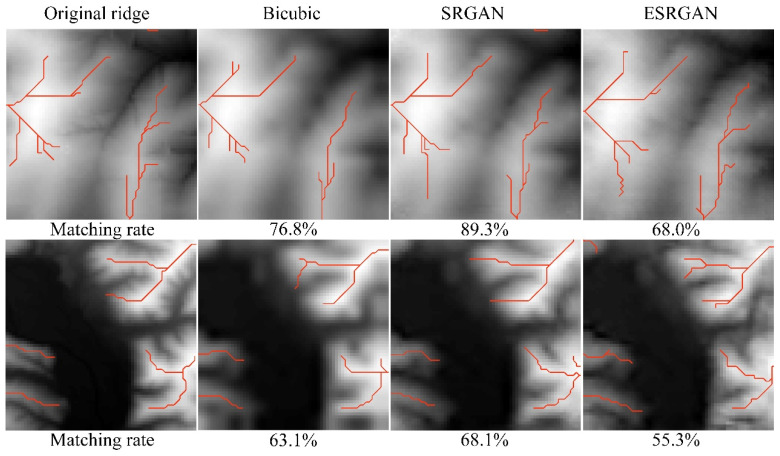
Ridges matching results of the selected methods.

**Figure 16:**
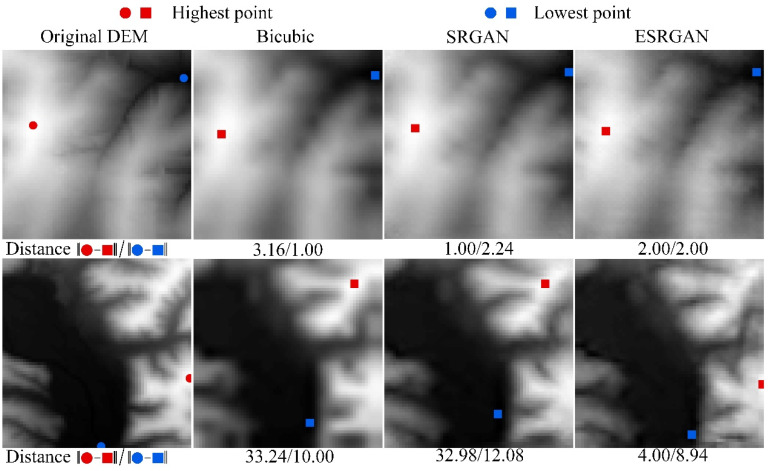
Results of displacement distances of critical points of the selected methods.

Figure 14, Figure 15 and Figure 16 present the results of structural lines (river and ridge) and critical points on the high-resolution DEMs generated by these methods, respectively. Firstly, it can be observed that SRGAN can obtain the best matching rate of river and ridge features. Then, ESRGAN outperforms bicubic interpolation in preserving river features, but bicubic interpolation performs better in preserving ridge features. As for the preservation of critical points (Figure 16), no obvious rule has been found in the two example results. In this regard, statistical results may be a better solution to evaluate the preservation of critical points, and the main conclusions can be obtained from Table 1 (please see Section 4.3.1). Therefore, to sum up, SRGAN outperforms the other methods in preserving most of the terrain features during DEM SR.

### 4.4. Ablation Experiments

#### 4.4.1. Quantitative Analysis of the Computational Performance

During the training process, all the experiments were implemented on the device with a single Nvidia RTX 2080Ti GPU and Intell Core i9-10900X CPU @ 3.70 GHz, and all the experiments are implemented using Python. The time and space costs required for training are listed in Table 2.

#### 4.4.2. Sensitivity of the Result on the Resolution

**Figure 17:**
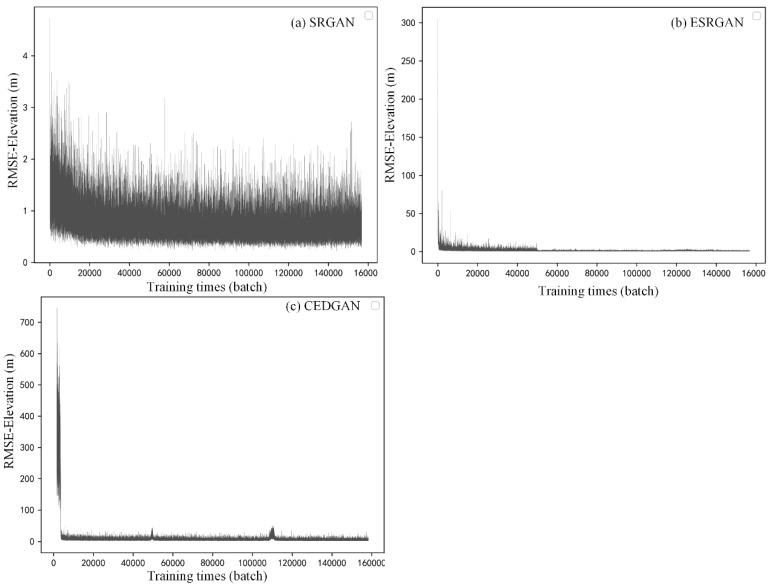
Training details of (**a**) SRGAN, (**b**) ESRGAN, and (**c**) CEDGAN based on the downsampling factor of 2.

Generally, recovering DEM from lower-resolution will bring greater challenges to the task of image SR or DEM SR, which means more details are required to be recovered. Most traditional image SR methods can recover natural images from 3× downsampling [19]. With the development of SR techniques, SRGAN firstly used a framework to recover photo-realistic images from 4× downsampling [17], and subsequent methods are proposed with the benchmark of 4× downsampling to demonstrate their robustness and effectiveness. Then, in this section, the impact of the resolution on different SR methods is investigated, and the downsampling factor is set as 2× for comparison. The training details and the obtained evaluation results based on testing data are presented in Figure 17 and Table 3, respectively. Firstly, considering the training phase (Figure 17), the average error of the generated high-resolution DEM obtained by SRGAN, ESRGAN, and CEDGAN gradually stabilized at 0.65 m, 0.73 m, and 5.51 m, respectively. From the results, the training error obtained in terms of 2× downsampling is lower than that of 4× downsampling (the corresponding errors in terms of 4× downsampling obtained by SRGAN, ESRGAN, and CEDGAN are 1.7 m, 2.2 m, and 6.4 m, respectively). Then, the evaluation results at the testing phase present a similar tendency (see Section 4.3.1). Therefore, it can be concluded that DEM SR is more difficult with the larger downsampling factors. Moreover, SRGAN still outperforms the other methods in terms of the RMSE-Elevation.

#### 4.4.3. Sensitivity of the Result on the Study Area

**Figure 18:**
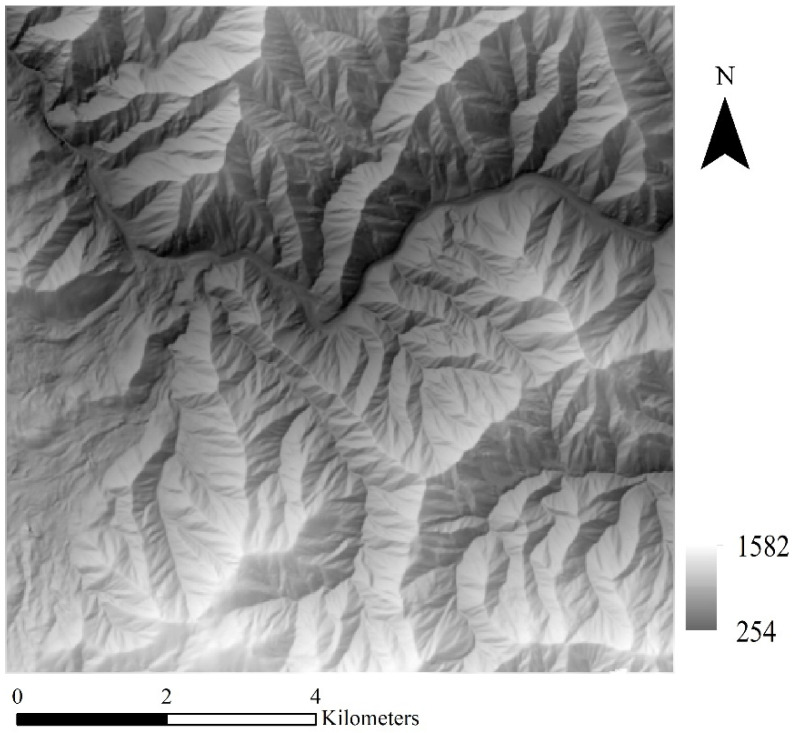
The DEM dataset used to test the sensitivity of the result on a new study area.

In the above experiments, we select DEMs from ten different regions including various terrains (e.g., areas of plateau mountain and basin) to test the robustness of different models. Usually, it is difficult for deep learning-based methods to deal with datasets they are not familiar with, which means the deep spatial features in a new area are difficult to be captured by a model trained with different datasets. Therefore, in this section, we want to explore how these models perform when faced with datasets in new domains. Therefore, the DEM dataset from a new area is used to test the sensitivity of the models. From Figure 18, the DEM contains complex terrain features with the size of 448 × 448, and the resolution is 20 m, which is also different from DEMs of 30 m resolution used in the above experiments. All the results are obtained by using the well-trained models in Section 4.2, and the results are listed in Table 4. The results present that bicubic outperforms other methods when dealing with such a new area. This suggests that although these deep learning models are trained with datasets from ten different regions, they are still weak to deal with new datasets. Then, compared with other deep learning methods, SRGAN still obtains the best results (with only one evaluation index exception), which also shows its robustness.

## 5. Conclusions and Future Work

SR is a classic topic in the field of computer vision, which can be widely applied in relevant tasks. This technique has attracted the attention of researchers from different fields for its easy understanding but important target, i.e., recovering high-quality texture details from lower-grade images. Nowadays, with the rapid development of machine learning (especially deep learning), image SR has made great progress. Although the task of DEM SR presents to be similar to image SR, there are few works that introduce image SR methods to DEM SR. The reason may be that DEM data have much more complex terrain features and larger elevation differences than natural images do. Moreover, the lack of DEM data for training may also be a limiting factor for previous research. Therefore, this paper attempts to investigate the performance of image SR methods when applied to DEM SR. More specifically, traditional bicubic interpolation method and three state-of-the-art SR methods (SRGAN, ESRGAN, and CEDGAN) based on neural networks are selected, and terrain-related evaluation indexes including slope, aspect, river, ridge, and critical points are used for comparison.

The results present that SRGAN outperforms the other three methods in terms of most evaluation indexes, and CEDGAN presents to be the worst one. Considering these two methods, there are two major differences. The first is that SRGAN designs a much deeper generator network than CEDGAN, and the second is that SRGAN uses specific loss functions and CEDGAN uses only adversarial loss. Network structure and loss function are also the most concerning points of the methods based on neural networks. Therefore, specifically designing the network structure and appropriate loss function for spatial data is an operative road to integrate GIS tasks and artificial intelligence (AI). Then, ESRGAN is proposed based on the adaptation of SRGAN, and ESRGAN is suggested to be able to generate natural images with better visual perception performance. Although it can be observed that many terrain features are learned by ESRGAN, these terrain features are always distorted. Therefore, ESRGAN is appropriate for natural image SR, but this learning process may not be suitable for DEM data. Finally, compared with methods based on neural networks, bicubic interpolation can output relatively balanced results considering different evaluation indexes. Moreover, this method can be applied to different terrain data without a training process, which is more efficient than learning-based methods. However, the main problem is that most of the terrain features are over-smoothed after the interpolation process. Therefore, for the DEM SR task, SRGAN is recommended when adequate training data and time are available, and bicubic interpolation can be used when efficiency rather than terrain features are concerned.

In terms of the results, we plan to extend our work from the following perspectives: (1) ESRGAN outperforms SRGAN in generating high-resolution natural images with realistic visual perception. Although the original design of ESRGAN is inappropriate for DEM SR, its idea of adapting SRGAN can be introduced to DEM SR, and that means specifically designed structures of neural networks and loss functions for terrain data have great potential to improve the effectiveness of terrain features preservation during DEM SR. (2) The idea of SR can also be introduced to other GIS fields. For example, trajectory points are usually collected between a fixed time interval (e.g., 1 min), which limits its application when dense trajectory points are needed [48]. Therefore, designing methods based on neural networks to increase the density of trajectory points is also a valuable exploratory direction.

## Figures and Tables

**Table 1:** Evaluation results of the fake high-resolution DEMs generated by the selected methods.

Evaluation Index	Bicubic	SRGAN	ESRGAN	CEDGAN
RMSE-Elevation	2.051	1.791	2.284	6.542
RMSE-Slope	3.615	3.284	3.762	7.000
ME-Slope	2.338	2.259	2.683	5.300
RMSE-Aspect	1.485	1.501	1.610	1.998
ME-Aspect	0.711	0.751	0.861	1.286
Distance-Peak Point	6.662	6.976	7.722	9.815
Distance-Valley Point	9.084	9.899	10.472	15.027

**Table 2:** The time and space costs required for training by the selected methods.

Evaluation Index	SRGAN	ESRGAN	CEDGAN
Time (h)	27.9	41.5	22.8
Space (MIB)	3375	4945	1110

**Table 3:** Evaluation results of the fake high-resolution DEMs generated by the selected methods based on the downsampling factor of 2.

Evaluation Index	Bicubic	SRGAN	ESRGAN	CEDGAN
RMSE-Elevation	0.701	0.684	0.768	5.351
RMSE-Slope	1.592	1.337	1.543	5.974
ME-Slope	0.884	0.867	0.981	4.407
RMSE-Aspect	0.933	1.089	1.090	1.848
ME-Aspect	0.309	0.425	0.423	1.121
Distance-Peak Point	5.247	5.763	5.716	8.678
Distance-Valley Point	7.636	8.557	8.448	13.691

**Table 4:** Evaluation results of the fake high-resolution DEMs generated by the selected methods based on a new study area.

Evaluation Index	Bicubic	SRGAN	ESRGAN	CEDGAN
RMSE-Elevation	7.433	9.648	18.882	88.236
RMSE-Slope	9.136	8.848	11.394	23.206
ME-Slope	5.893	6.185	8.079	19.087
RMSE-Aspect	1.280	1.315	1.411	2.064
ME-Aspect	0.554	0.591	0.667	1.384
Distance-Peak Point	6.026	7.121	6.441	9.726
Distance-Valley Point	7.367	9.075	10.742	13.379

## Data Availability

This study makes use of third-party code sources from SRGAN [17], ESRGAN [18], and CEDGAN [28]. Our codes are based on the original revision of SRGAN, ESRGAN, and CEDGAN, and basic codes are revised to enable these models to apply to DEM data. The data and codes that support the findings of this study are available in [figshare.com] with the identifiers (https://figshare.com/s/3fe0b1313938b0db994a (accessed on 13 May 2021)).
